# Early experience with the SmartGUIDE: A new generation of in-situ deflectable 0.014-inch guidewire

**DOI:** 10.1177/15910199251399461

**Published:** 2025-11-27

**Authors:** Mohammad Al-Tibi, James Lord, Shah Islam, Federico Carpani, Eef J Hendriks, Emily Chung, Alexandre Boutet, Ronit Agid, Zeev Itsekzon Hayosh, Pascal J Mosimann

**Affiliations:** 1 563025 Toronto Western Hospital Division of Neuroradiology, Joint Department of Medical Imaging, Toronto, ON, Canada; 2 195157 The Walton Centre for Neurology and Neurosurgery, Neuroradiology Division, Liverpool, UK

**Keywords:** Guidewire, innovation, arcuable, deflectable, steerable

## Abstract

**Background:**

Neurointerventional procedures are increasingly complex, requiring access to more distal vasculature. SmartGUIDE 0.014-inch guidewire (Artiria Medical, Geneva, Switzerland) is an FDA-approved dynamic deflectable-tip guidewire, manipulated by an external handle to lock the distal end, allowing active shaping of micro/balloon catheters. We report the first clinical experience using the SmartGUIDE in various neurovascular interventions.

**Methods:**

Neurointerventional procedures utilizing the SmartGUIDE from May 2024 to July 2025 were retrospectively analyzed. Clinical outcomes, technical success, and periprocedural complications were assessed. The primary endpoint was successful delivery of a microcatheter tip using the SmartGUIDE to the predefined target without the use of an adjunctive device or microwire. Procedures requiring alternative microwires to replace SmartGUIDE were considered unsuccessful.

**Results:**

The SmartGUIDE was used in 25 procedures: aneurysm embolization (*n = *9), arteriovenous malformation/fistula embolization (*n = *5), tumor embolization (*n = *4), venous and carotid stenting (*n = *4), balloon test occlusion (*n = *2) and endovascular thrombectomy (*n = *1). SmartGUIDE enabled access to challenging neurovascular targets, successfully reaching the target vessel independently in 24/25; 96% of cases. No device-related complications incurred, such as perforation or dissection. All patients were discharged at their baseline clinical status, except the EVT patient who improved by seven NIHSS points.

**Conclusions:**

SmartGUIDE's deflectable-tip wire achieved high technical success and proved safe across various neurointervention. SmartGUIDE improves microcatheter navigation without repeated ex-vivo tip shaping, adjunct tools, or looping through aneurysms. Locking the SmartGUIDE tip can deflect microcatheters and prevent them from herniating into their parent vessel during side branch navigation. Larger studies are needed to evaluate effectiveness across broader clinical indications.

## Introduction

Guidewires are essential in accessing and crossing vascular structures and delivering interventional tools.^1–3^ Early guidewires were simple and straight; the introduction of different segments and hand-shapeable tips in the 2000s improved navigation in tortuous vasculature. Still, modern guidewires often require multiple reinsertions and manual shaping, which diminishes flexibility, risks structural damage, and reduces efficiency.^2–4^ As wires advance through tortuous vessels, tip control decreases,^5^ reducing success rate to reach distal targets.^3^ It was estimated that up to 20 extractions or insertions may be required per procedure,^6^ raising the risk of thromboembolism, vessel perforation, radiation exposure, infection, and equipment waste.^3,6^

**Figure 1.**
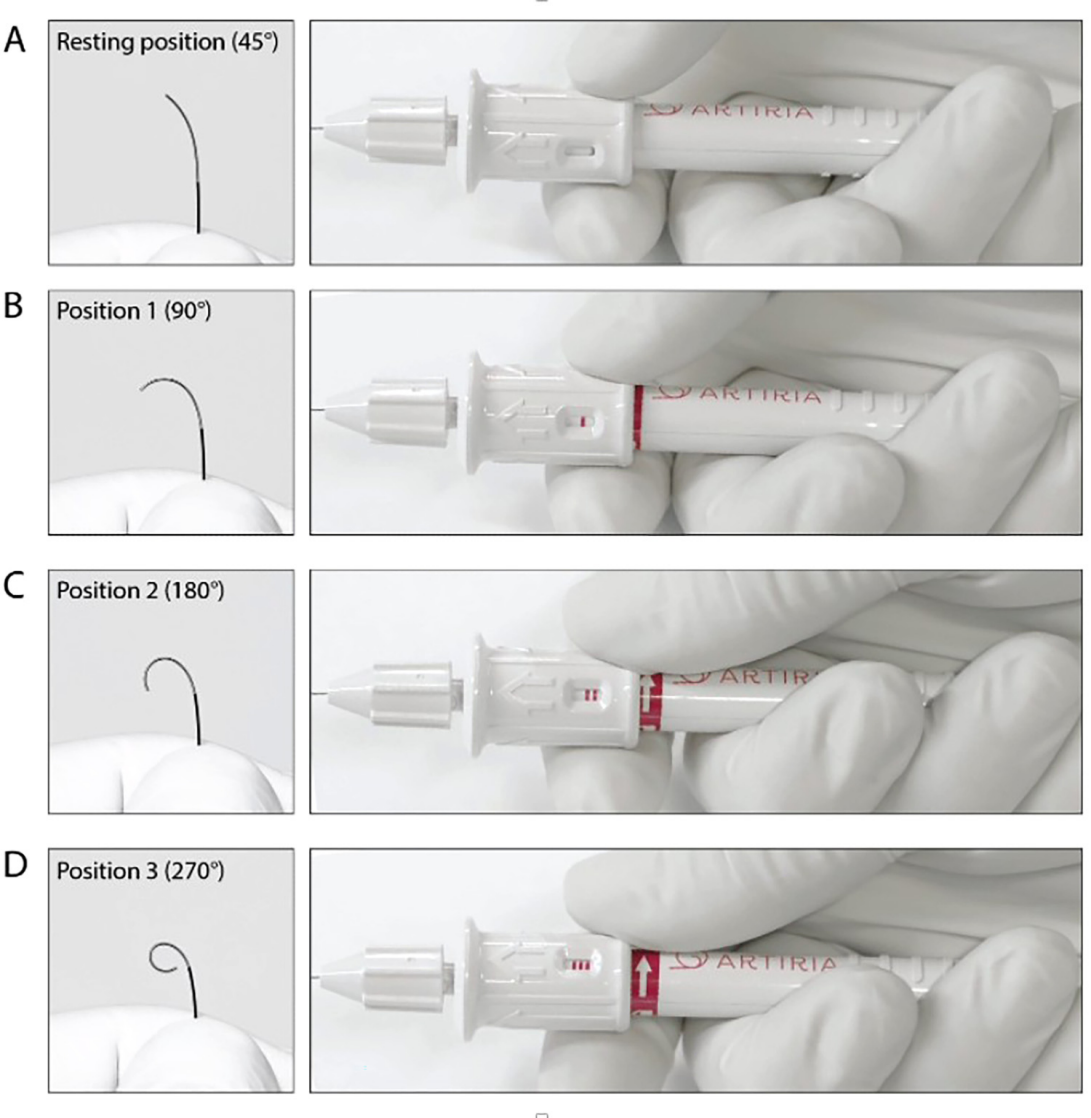
SmartGUIDE guidewire and handle components. The actuator allows the physician to control the tip deflection angle by turning the pusher across the four different tip positions A–D.

SmartGUIDE introduces dynamic, single-handed tip deflection during navigation. Controlled via a proximal handle, the wire can be shaped and torqued in real-time (Figure 1). It maintains optimized pushability, torqueability, support, and radiopacity across its length. Designed for use with 0.0165–0.021-inch inner lumen micro- or balloon catheters, SmartGUIDE enhances distal access without removal and reshaping of the wire. It is currently the only FDA-approved 0.014-inch deflectable tip neuro-interventional wire available on the market.

Given these novel features, we sought to gain initial clinical experience with SmartGUIDE in a broad spectrum of neurointerventional procedures. We hypothesized that SmartGUIDE could be safely and effectively applied across diverse indications, enabling reliable target vessel access while maintaining a favorable safety profile.

The objective of this study is to evaluate the feasibility, technical performance, and safety of the SmartGUIDE deflectable-tip microwire across a range of neurointerventional procedures.

## Methods

### Ethics approval and patient selection

With institutional research ethics board approval and patient consent, we retrospectively analyzed all cases performed at a single tertiary level neurointerventional center using SmartGUIDE over a 15-month period between May 2024 and July 2025 under Canada Health's special access program.

Inclusion criteria included all adults (≥18 years) undergoing any elective or emergency neurointervention with SmartGUIDE as the primary microwire, selected by a senior operator with >15 years’ experience who was thoroughly trained on silicone models. Exclusion criteria included age <18, lack of consent, or anticipated need for more than one guidewire.

Collected data included demographics, medications, procedural details (e.g., intervention performed, indication, access site, devices used and end of procedure angiographic outcome. Device-related complications were defined as any intra-operative dissection, vasospasm, perforation, or intracranial hemorrhage attributable to the SmartGUIDE wire, confirmed by fluoroscopy or intra-operative cone-beam CT imaging. Neurological status was evaluated via modified Rankin Scale (mRS) at admission/discharge. Ninety-day mRS was not captured as it was determined it would not be attributable to late adverse outcomes due to wire usage during the intervention.

Technical success was defined as delivering a microcatheter to the target with a single SmartGUIDE wire. Technical failure was defined as inability to reach defined target and/or unplanned replacement with another microwire.

### Procedure details

Preoperative imaging included computed tomography (CT), computed tomography angiography (CTA), magnetic resonance imaging (MRI), or time-of-flight magnetic resonance angiography (TOF-MRA). Procedures were conducted on a Philips Allura Clarity biplane suite. Twenty-two patients underwent general anesthesia; three had local anesthesia with or without conscious sedation.

Antiplatelet regimen comprised Aspirin 75 mg and Clopidogrel 75 mg OD or Ticagrelor 90 mg BID for five days pre-procedure whenever appropriate. P2Y12 inhibition is not tested peri-procedurally in our institution.

Vascular access was performed under ultrasound guidance (radial, femoral, jugular, or combined). Closure devices (Angioseal, Terumo) were used for all arterial punctures whilst venous punctures were managed by manual compression.

Heparin boluses (70–100 IU/kg, +1000 IU/h) were used in all but the endovascular thrombectomy cases. All trans-radial cases received intra-arterial verapamil and nitroglycerin following sheath administration.

Triaxial/biaxial platforms with appropriate catheters were used. SmartGUIDE was the primary 0.014-inch wire in all these consecutive cases.

On-table post-procedure cone-beam CT was systematically acquired to exclude immediate hemorrhagic complications. Follow-up imaging depended on the intervention type. Aneurysm embolization and venous stenting cases were followed up with contrast-enhanced MR or CT venography at 3 months. Carotid stenting and EVT procedures were followed up with non-contrast head CT with or without CTA at 24 h. Tumor embolizations were followed with postoperative contrast-enhanced MRI.

### SmartGUIDE device details

**Figure 2.**
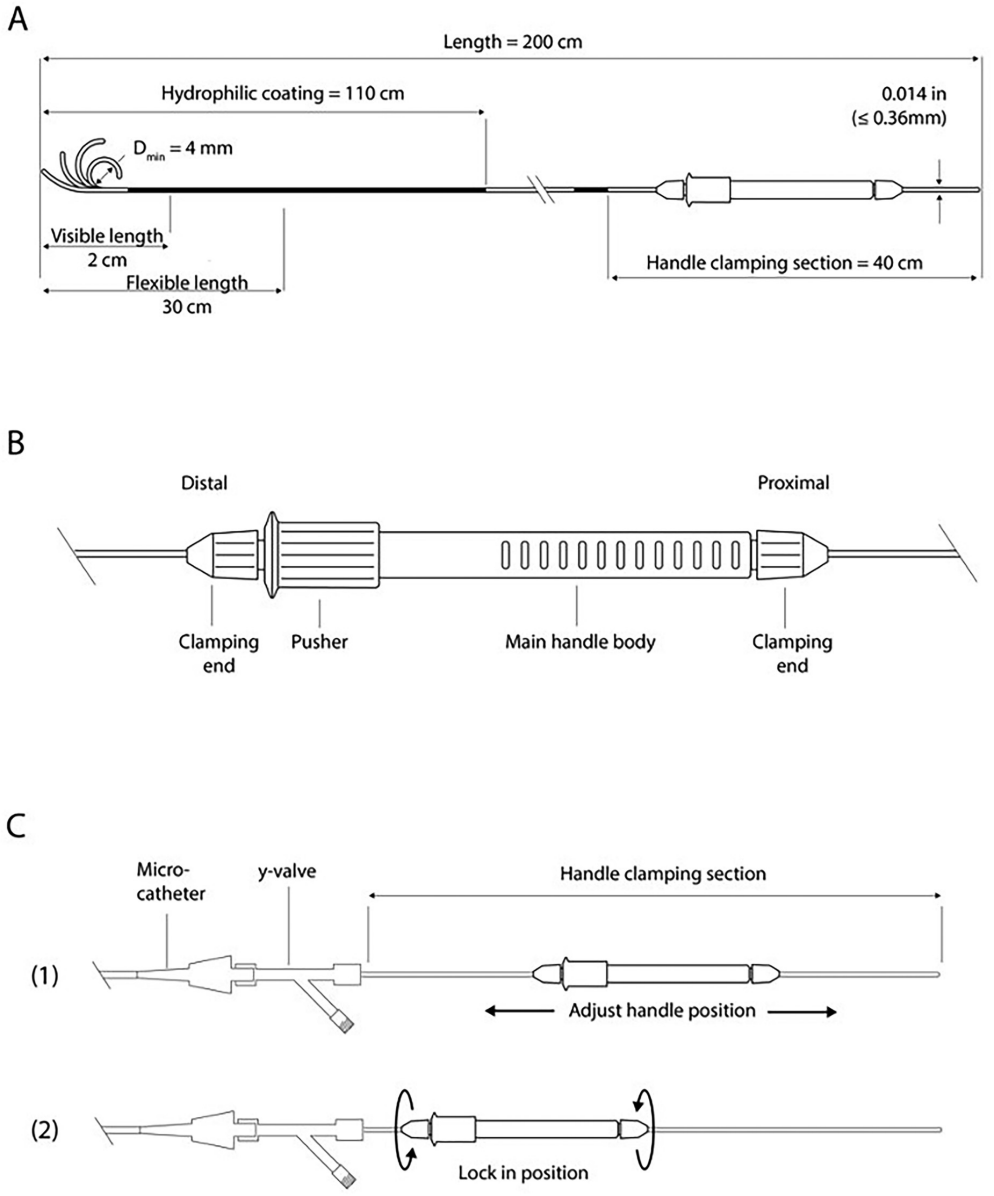
SmartGUIDE wire and actuator handle components. Schematic overview of the specifications of the SmartGUIDE microwire (A) and the actuator (B and C).

SmartGUIDE is a 0.014-inch, 200-cm steerable wire with a deflectable tip and a proximal handle for single-handed control and torque (Figure 2). The tip can be locked in position to shape a microcatheter and then rotated and torqued from within to direct the microcatheter tip itself. The wire tip can also be maximally deflected ahead of the microcatheter and then retracted to bend the microcatheter tip which can then be advanced without having to lead with the tip of the wire, to avoid getting stuck in small side branches.

The proximal handle is detachable and repositionable, which allows the physician to utilize the most appropriate working wire length or to use a docking exchange length wire. An exchange-length version of SmartGUIDE and a robotic tip-shaping module are in development.

### Clinical outcomes and follow-up

Clinical outcomes were assessed using specific and general measures. Specific outcomes included technical performance, such as SmartGUIDE's ability to reach the target lesion, and safety measures, monitoring device-related complications like perforation, dissection, thromboembolism, infection, and vasospasm. General outcomes involved complications unrelated to the wire, such as symptomatic intracranial hemorrhage (symptomatic intracranial hemorrhage [sICH]), access site issues, and discharge status. sICH was classified per ECASS II, defined by an NIHSS increase ≥4 points or death within 24 h.^7^ In the EVT case, angiographic outcomes were measured using the expanded Thrombolysis in Cerebral Infarction (eTICI) scale, with successful recanalization defined as eTICI ≥2b. Pre-stroke functionality was assessed via the mRS, and NIHSS was recorded at admission and discharge.

## Results

A total of 25 patients (18 females and 7 males) with a median age of 62 years (20–79 years) underwent endovascular treatments using SmartGUIDE. Procedures included: Balloon/stent-assisted aneurysm embolization (*n = *9), dural arteriovenous fistula embolization (*n = *4), arteriovenous malformation embolization (*n = *1), tumor embolization (*n = *4), venous manometry and stenting (*n = *3), balloon test occlusion (*n = *2), endovascular thrombectomy (*n = *1), and carotid stenting (*n = *1).

Vascular access was heterogeneous. Seven patients required more than one access site. Radial access was used in 16 cases, femoral arterial in nine cases, femoral venous in two cases, and jugular venous in four cases.

Table 1 demonstrates an overview of neurointerventional procedures performed using SmartGUIDE. SmartGUIDE successfully reached its target access site independently in 24 out of 25 cases (96%) without requiring removal or manual tip shaping. One case did not achieve independent target access and required the use of a second microwire in conjunction with SmartGUIDE to reach the targeted vessel. This involved an indirect carotid-cavernous fistula that posed significant challenges due to the need for retrograde venous navigation from the external jugular vein through the facial and angular veins to reach the superior ophthalmic vein and the cavernous sinus. Although SmartGUIDE advanced well initially, it could not form the necessary S-shaped configuration to navigate the angular vein and was temporarily replaced with a Synchro 14-inch microwire (Stryker). After this segment was crossed, SmartGUIDE was reintroduced and successfully used to access the fistulous portion of the cavernous sinus. The Synchro wire was unable to navigate this segment due to a recurrent angle that required the locked, arcuated tip of SmartGUIDE to steer the microcatheter for effective embolization. In five cases (20%), SmartGUIDE served as a support wire for balloon catheter delivery (with docking exchange wire used in one case). In two cases (8%), the SmartGUIDE wire showed proximal kinks post-removal due to overtightened handles, which did not negatively impact wire manipulation during the procedures. The predefined target was reached in both cases and did not necessitate wire exchange. No intracranial device-related complications such as hemorrhage, thrombosis, dissection or vasospasm were encountered. Access site hematoma was encountered in two cases (8%), not attributed to the SmartGUIDE. Elective cases (*n = *21) were discharged within 48 h with no new deficits. Acute and semi-acute cases (*n = *4) had longer hospital stays but had no neurological worsening.

**Table 1.** Interventional procedures performed using SmartGUIDE.

Patient number	Intervention performed	Indication	Access site	Use of SmartGUIDE	Name of target vessel. Has it reached independently or in conjunction with another microwire?	Microcatheter used with SmartGUIDE	Device deployed	End of procedure angiographic outcome	Intra-operative Adverse Events
1	Embolization of ophthalmic artery	Tumor, Retro-orbital myofibroma.	Arterial trans-radial	Treatment delivery	Ophthalmic artery – Independently	Headway Duo	Coils	Near total tumor devascularization	No
2	Coiling, stent assisted	SCA aneurysm, unruptured.	Arterial trans-radial	Supporting wire	SCA – Independently	Headway Duo	N/A (headway duo was used for vessel protection)	Aneurysm occlusion, mRR 1	No
3	Coiling, stent assisted	ACOM aneurysm, unruptured.	Arterial trans-femoral	Treatment delivery	ACOM aneurysm – Independently	Echelon 10	Coils	Aneurysm occlusion, mRR 1	No
4	Balloon test occlusion	ICA aneurysm (paraophthlamic), unruptured	Arterial bilateral trans-femoral	Supporting wire	Paraophthalmic ICA – Independently	Eclipse DL balloon	N/A	Successful BTO (Pass)	No
5	Coiling, stent assisted	ACOM aneurysm, unruptured	Arterial trans-radial	Treatment delivery	ACA A2 – Independently	Eclipse DL balloon	Leo stent	Aneurysm occlusion, mRR 1	No
6	Endovascular thrombectomy	Acute ischemic stroke, MCA M1 segment.	Arterial trans-femoral	Treatment delivery	MCA M2 – Independently	Trevo Track 21	RED 72 Solitaire stent retriever	TICI 3	Yes – Groin hematoma
c	Venous manometry and stenting	Venous stenosis, transverse sinus	Arterial trans-radial and venous trans-jugular	Treatment delivery	Venous sinuses, crossing the torcula– Independently	Rebar 18	Carotid Wallstent	Reduced venous pressure gradient	No
8	Embolization of bilateral MMA	Tumor, parietal meningioma	Arterial trans-femoral	Treatment delivery	Bilateral MMA – Independently	Echelon 10	PVA Particles – 45–150 microns	Reduced tumor flow	No
9	Carotid angioplasty and stenting	Carotid artery web (Symptomatic)	Arterial trans-radial	Treatment delivery	Petrous ICA – Independently	Aviator PTA balloon 4 × 20	Carotid Wallstent	Improved vessel caliber	No
10	Embolization of PICA distal branches	Tumor, cerebellar hemangioblastoma	Arterial trans-radial	Supporting wire	Distal PICA – Independently	Headway Duo	n-BCA	Reduced tumor flow	No
11	Balloon assisted coiling	PCOM aneurysm, unruptured	Arterial trans-radial	Treatment delivery	PCOM aneurysm – Independently	Echelon 10	Coils	Aneurysm occlusion, mRR 1	No
12	Transvenous embolization	dAVF, Indirect CCF, high grade	Arterial trans-radial and venous trans-femoral	Treatment delivery	Cavernous sinus (through facial vein/SOV) - In conjunction with Synchro 14 microwire	Headway Duo	Onyx	Reduced flow into the dAVF	No
13	Balloon assisted coiling	ACOM aneurysm, unruptured	Arterial trans-radial	Treatment delivery	ACOM aneurysm – Independently	Echelon 10	Coils	Aneurysm occlusion mRR 2a	No
14	Coiling, stent assisted	ACOM aneurysm, unruptured	Arterial trans-radial	Treatment delivery	ACOM aneurysm – Independently	Headway Duo	Coils	Aneurysm occlusion mRR 1	No
15	Trans-arterial embolization	dAVF, transverse sinus.	Arterial trans-radial	Treatment delivery	Parietal MMA – Independently	Headway Duo	Onyx	Complete dAVF occlusion	No
16	Transvenous embolization	dAVF, Indirect CCF, high grade	Arterial trans-radial and venous trans-jugular	Treatment delivery	Cavernous sinus (through Inferior petrosal sinus) – Independently	Headway Duo x 2	Coils and Onyx	Reduced flow into the dAVF	No
17	Trans-arterial embolization	AVM, scalp	Arterial trans-femoral	Supporting wire	Posterior auricular artery – Independently	Scepter XC balloon (reflux protection)	N/A	Reduced flow into the AVM	No
18	ICA stenting	ICA aneurysm (cavernous), giant, unruptured	Arterial trans-femoral	Treatment delivery	Paraophthalmic ICA – Independently	Excelsior XT-27	Bentley BeGraft covered stent	Aneurysmal flow stasis	No
19	Venous manometry and stenting	Venous stenosis, transverse sinus	Arterial trans-radial and venous trans-jugular	Treatment delivery	Venous sinuses, crossing the torcula– Independently	Rebar 18	Carotid Wallstent	Reduced venous pressure gradient	No
20	Embolization of lingual artery pseudoaneurysm	Tumor, tongue SCC.	Arterial trans-femoral	Treatment delivery	Lingual artery – Independently	Headway Duo	n-BCA	Occlusion of pseudoaneurysm	No
21	Stent-assisted coiling	PICA aneurysm, unruptured	Arterial trans-femoral	Treatment delivery	PICA aneurysm – Independently	Headway Duo	Coils	Aneurysm occlusion, mRR 2a	No
22	Stent-assisted coiling	SMA aneurysm, unruptured	Arterial trans-radial	Supporting wire	Distal SMA – Independently	Eclipse DL balloon	N/A	Aneurysm occlusion, mRR 1	Yes – Wrist hematoma
23	Venous manometry and stenting	Venous stenosis, transverse sinus	Arterial trans-radial and venous trans-jugular	Treatment delivery	Venous sinuses, crossing the torcula– Independently	Excelsior XT-27	Carotid Wallstent	Reduced venous pressure gradient	No
24	Transvenous embolization of dural arteriovenous fistula	dAVF, Indirect CCF, low grade (symptomatic)	Arterial trans-radial and venous trans-femoral	Treatment delivery	Cavernous sinus (through IPS) – Independently	Headway Duo	Coils and onyx	Complete dAVF occlusion	No
25	Balloon test occlusion and ICA stenting	Cochlear dehiscence over petrous ICA causing pulsatile tinnitus	Arterial trans-femoral	Treatment delivery	Paraophthalmic ICA – Independently	Eclipse DL balloon	N/A	Improved angiographic appearances	No

ACOM: Anterior communicating artery; AVM: Arteriovenous malformation; BTO: Balloon test occlusion; CCF: Carotid-cavernous fistula; DL: Double lumen; dAVF: Dural arteriovenous fistula; IPS: Inferior petrosal sinus; ICA: Internal carotid artery; MCA: Middle cerebral artery; MMA: Middle meningeal artery; mRR: Modified Raymond-Roy classification; n-BCA: n-Butyl cyanoacrylate; PICA: Posterior inferior cerebellar artery; PCOM: Posterior communicating artery; PTA: Percutaneous transluminal angioplasty; SCA: Superior cerebellar artery; SCC: Squamous cell carcinoma; SG: SmartGUIDE; SOV: Superior ophthalmic vein; TICI: Thrombolysis in cerebral infarction.

Compatible microcatheters used in our series ranged from 0.0165 to 0.027 inches in inner diameter. These included, in order of frequency of use: Headway Duo (Terumo Neuro), Echelon 10 (EV3, Medtronic), Eclipse dual-lumen balloon (Balt), Rebar-18 (EV3, Medtronic), Excelsior XT-27 (Stryker), Scepter XC balloon (Terumo Neurovascular), and Trevo Trak 21 (Stryker). In addition, the Aviator Plus percutaneous transluminal angioplasty (PTA) Balloon Dilatation Catheter (Cordis) and the Carotid Wallstent Monorail (Boston Scientific) were successfully delivered over SmartGUIDE. No compatibility issues were encountered.

The most common intervention performed with SmartGUIDE was unruptured aneurysms (*n = *9). SmartGUIDE facilitated various embolization techniques, including balloon- or stent-assisted coiling and flow diversion. Most cases required more than one microcatheter; typically, one for coiling and another for balloon or stent delivery. In coiling procedures, the tip was advanced into the aneurysm in a medium or high deflection configuration (position I or II, respectively) to minimize the risk of iatrogenic perforation. In one case (Patient 22, Table 1), SmartGUIDE was used in the management of a non-operable giant superior mesenteric artery aneurysm. The vascular configuration closely resembled that of a basilar tip or MCA bifurcation aneurysm. SmartGUIDE enabled direct access to the efferent side branches without the need to loop the microwire or microcatheter around the aneurysm dome, an infrequent albeit hazardous maneuver.

In dural arteriovenous fistulae (*n = *5), SmartGUIDE was used to navigate the 0.0165-inch headway duo microcatheter to distal targets: The middle meningeal artery for a transverse sinus fistula (Patient 15), and the cavernous sinus in CCFs via the inferior petrosal sinus (Patients 16, 24) or the superior ophthalmic vein via the facial vein (Patient 12). Implants included coils or liquid embolic agents such as Squid (Balt) or Onyx (Medtronic).

Tumor embolization cases (*n = *4) were performed in both elective and emergent pre-operative settings. SmartGUIDE enabled successful cannulation of small-caliber vessels with sharp angulations, such as the ophthalmic artery (Patient 1) and the posterior inferior cerebellar artery (Patient 10), both of which featured a proximal 180-degree curve. These vessels are typically challenging to access using standard guidewires and often require ancillary devices like balloons. In contrast, SmartGUIDE achieved access by dynamically adjusting its tip across three deflection settings, eliminating the need for additional support devices.

The EVT case in our cohort achieved eTICI 3 recanalization in one pass using combined aspiration and single stentretriever, with a groin-to-reperfusion time of 24 min. The SmartGUIDE wire was used to cross the long hyperdense thrombus in a high deflection tip configuration (position III). No wire perforation occurred. At discharge, the patient's NIHSS score improved from 10 to 3, and baseline functional independence (mRS 1) was maintained.

## Discussion

SmartGUIDE's real-time deflection enabled successful completion of diverse procedures using a single wire. Its safety profile appears comparable to conventional 0.014-inch wires in these first 25 consecutive patients. The ability to dynamically shape and torque microcatheters offers a novel approach to navigating bifurcation branch points.

### Illustrative cases

**Figure 3.**
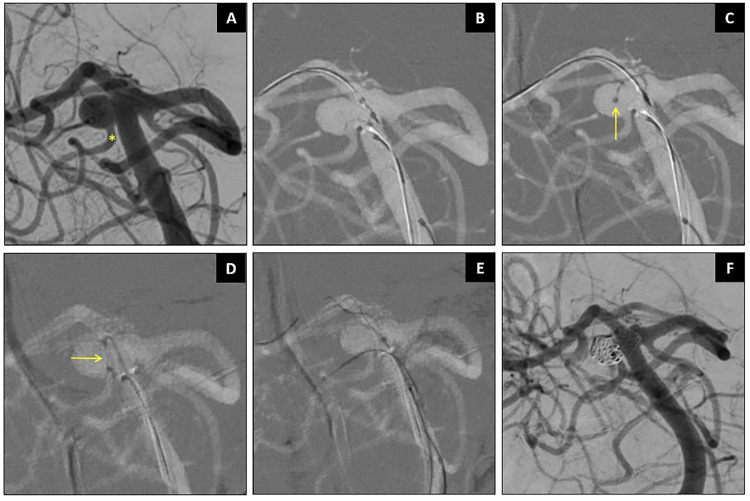
Stent-assisted coiling of right SCA aneurysm using SmartGUIDE. Caption: Wide-necked right SCA aneurysm with ipsilateral SCA arises from the aneurysm neck (asterisk in A). The SmartGUIDE could cannulate the right SCA by locking it in a “U Shape” before sliding the microcatheter (arrows) over the wire (B–D). The SCA remained patent (F).

**Figure 4.**
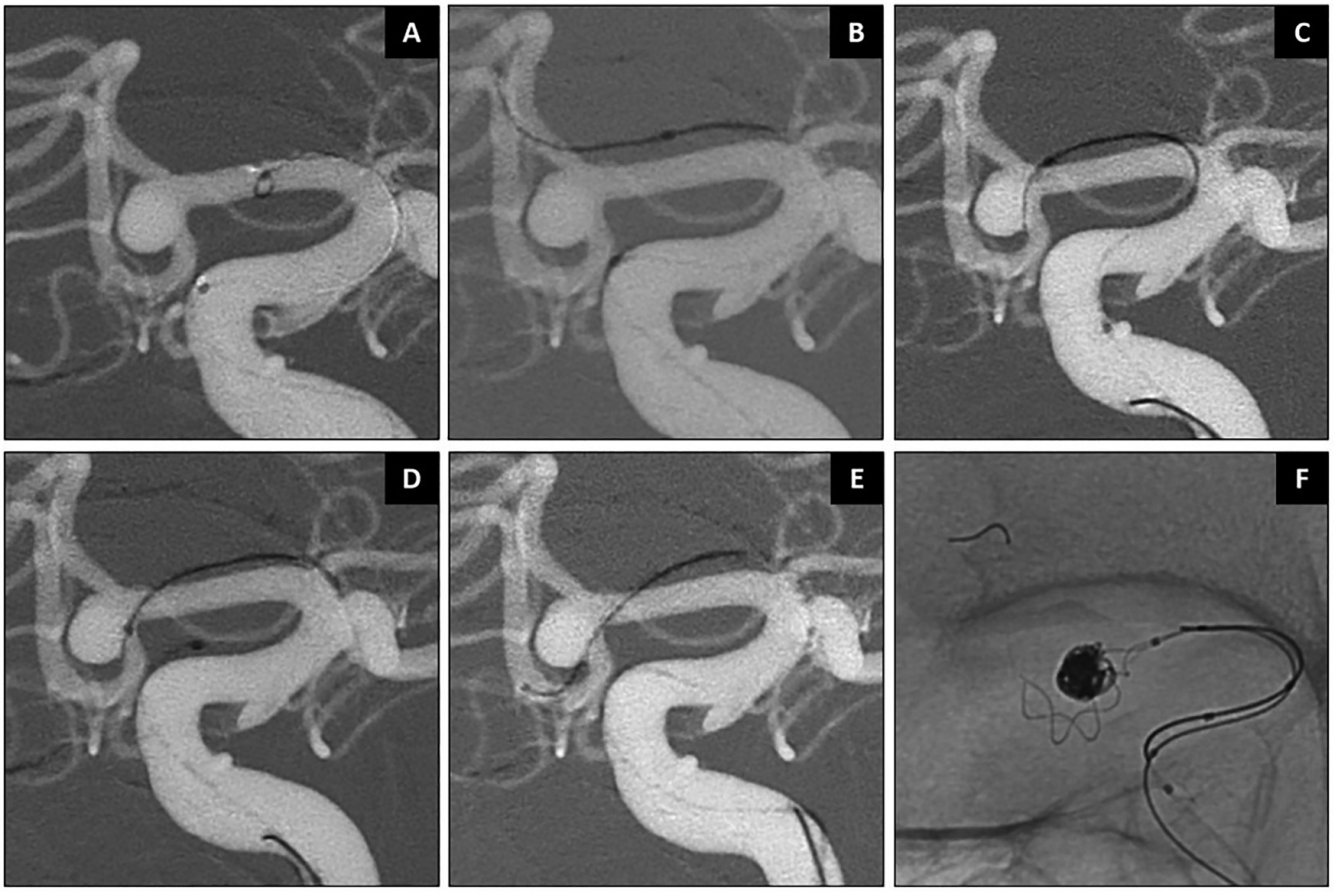
Stent-assisted coiling of ACOM aneurysm using SmartGUIDE. Working projections shown (A). The SmartGUIDE microwire was used to cannulate the contralateral ACA A2 and offer stent assisted coiling using a braided stent (B–F).

**Figure 5.**
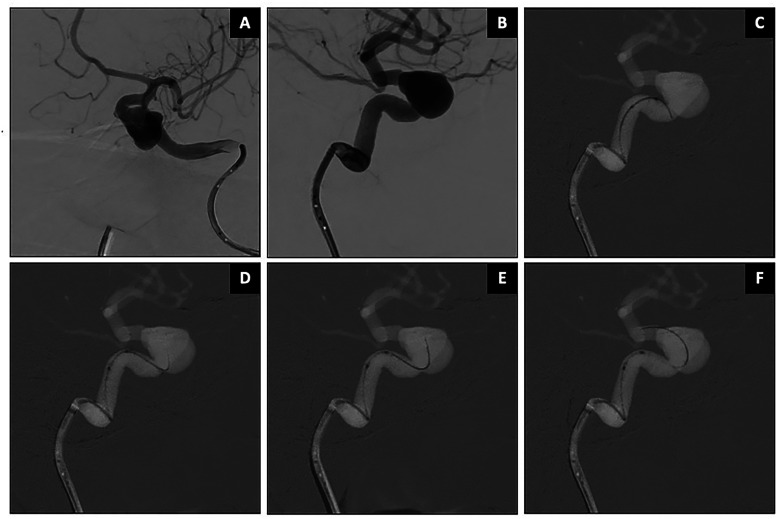
Balloon test occlusion on an unruptured large ICA aneurysm using SmartGUIDE. AP and lateral views (A–B) show the working projections. SmartGUIDE microwire was could navigate the aneurysm inflow and outflow by adjusting the tip positions (C–F).

**Figure 6.**
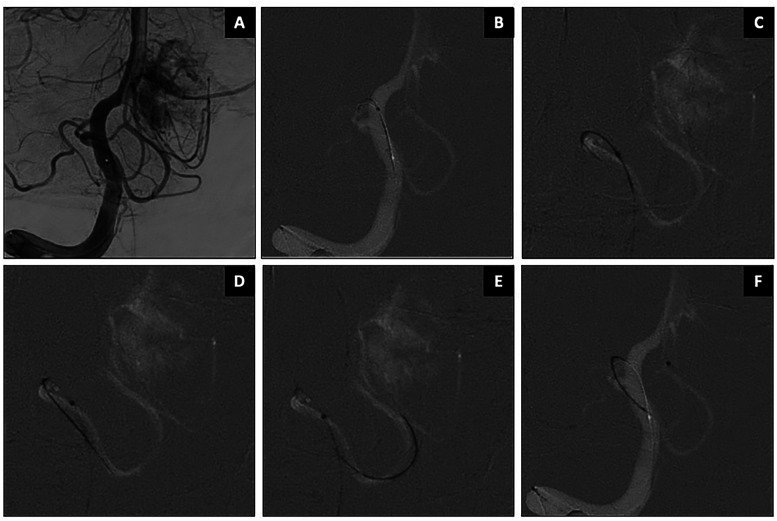
Pre-operative embolization of posterior fossa tumor supplied by right PICA branches. AP working projection shown in panel (A). SmartGUIDE wire was used to safely navigate through the PICA's proximal 180-degree curve as well as its distal tortuous course (B–F).

- Case 1: Stent-assisted coiling of a right SCA aneurysm (Patient 2, Figure 3). The SCA originated from aneurysm neck, with a sharp 180° turn. Accessing this vessel with a conventional non-steerable microwire typically requires maneuvers such as looping the wire into the aneurysm dome, especially in large or partially thrombosed aneurysms. However, the SmartGUIDE wire successfully cannulated the right SCA by locking into a “U-shape” or “mini-Simmons” configuration, allowing the microcatheter to slide over the wire without the need to loop it into the aneurysm dome or to push it far inside the SCA.
- Case 2: Stent-assisted coiling of an ACOM aneurysm (Patient 3, Figure 4). The SmartGUIDE's functionality in navigating the sharp angle of the contralateral A2 branch was achieved by first locking the device into a “J-shape” and sliding the Echelon 10 microcatheter over the wire along the A1 segment, then reducing the tip into an “L-shape” configuration dynamically to cross the ACOM. This approach allowed the operator to navigate the devices safely before deploying the braided stent and coils.
- Case 3: Balloon test occlusion was performed in consideration of parent vessel sacrifice for a large cavernous ICA aneurysm (Patient 4, Figure 5). The interaction between the SmartGUIDE wire and the Eclipse double-lumen balloon (Balt) facilitated tip deflection and navigation across both the afferent and efferent vessels, without the need to loop the wire into the aneurysm dome to access the efferent branch.
- Case 4: Pre-operative embolization of a hypervascular cerebellar tumor supplied by bilateral PICA branches (Patient 10, Figure 6). SmartGUIDE was used to navigate both the PICA's proximal 180-degree curve and its distal tortuosity by dynamically adjusting the tip's shape between the three curve settings.
- Case 5: Pre-operative embolization was performed for a hypervascular retro-orbital tumor supplied by the ophthalmic artery (Patient 1, Table 1). The ophthalmic artery arose from the internal carotid artery at an acute angle. Although catheterization with a conventional, non-steerable microwire is feasible, it typically requires the use of adjunctive devices - such as a balloon catheter positioned distal to the ophthalmic artery ostium - to facilitate redirection of the microwire into the vessel. In this case, the SmartGUIDE microwire enabled selective catheterization of the ophthalmic artery by allowing dynamic intra-operative modulation of the wire tip configuration, thereby directing the microcatheter through the vessel without the need for additional adjunctive devices. This case has been previously illustrated in a published technical video by our group.^8^
- SmartGUIDE enabled Carotid Wallstent deployment for venous and arterial stenosis (Patients 7, 9, 12, and 23; Table 1). The Traxcess 0.014-inch docking wire (Microvention, Terumo Neuro) was required to extend the SmartGUIDE in one venous stenting case following microcatheter manometry.

### Comparison to other real-time steerable microwires/devices

A retrospective study on the 0.014-inch Columbus/Drivewire (Rapid Medical, Israel) in 36 patients demonstrated similar features of a real time steerable microwire.^9^ Despite early enthusiasm, the company retrieved it from the market due to its poor torqueability, visibility and high propensity to kink, as well as failure to perform in tortuous anatomy.

The second-generation Drivewire 24 is a 0.024-inch steerable guidewire with a proximally controlled tip that enables intravascular steering for selective navigation of diagnostic or therapeutic catheters.^10^ Early clinical experience has shown high technical success across various neurointerventional procedures. However, its larger diameter restricts use to microcatheters with an inner diameter >0.024 inches. In contrast, the SmartGUIDE 0.014-inch wire supports a broader range of smaller and medium microcatheters, enabling access to further distal and/or narrower targets, as shown in our series.

The recently introduced Gama Microchap microcatheter (Balt), launched in Essen, Germany, was formally announced in WFITN in January 2025.^11^ It features a pre-shaped Simmons curve designed to facilitate access to sharply angulated arteries near the aneurysm neck. It is particularly useful for engaging difficult branches in wide-neck or branching aneurysms. Microchap is available in two Simmons curve configurations, though no studies have been published to date. SmartGUIDE can be used to configure microcatheters in a mini-Simmons shape, as shown in Figure 3, which broadens the options to match each operator's preferred material, providing greater flexibility than fixed-shape microcatheters.

The Bendit Microcatheter (Petah Tikva, Israel) is a 0.021–0.027 inch microcatheter with three-dimensional controlled bending and torque capabilities, usable with or without a guidewire. It received FDA clearance in 2019, with early clinical experience in neurointerventional procedures reported by Killer-Oberpfalzer et al.^12^ and Qiao et al.^13^ The company mentioned it was evaluating a 0.017″ microcatheter to potentially deliver cage-like implants in side-wall aneurysms which are notoriously challenging to treat with such implants although no further clinical experience has been reported since.

The Artiria Medical SmartGUIDE 0.014-inch guidewire is compatible with 0.0165–0.027inch lumen catheters, as shown in Table 1. Our experience suggests that SmartGUIDE can be dynamically shaped to navigate highly angulated branches and offers capabilities that classic microwires cannot. When fully arcuated (position III), the wire can be pulled back into the microcatheter or “railed in,” allowing it to deflect the tip of the microcatheter into various shapes, including U or “simmons-like” configurations. The catheter can subsequently be torqued by rotating the locked wire from within and its angulation dynamically changed to a lesser angle by reducing the arcuation to position II or I. Moreover, the fixed arcuated wire can be used to slide the microcatheter directly into a branch without having to lead with the wire. This holds the catheter and tends to prevent it from prolapsing into the aneurysm when forward pressure is incrementally applied. Once locked inside the microcatheter, the wire can be relaxed to a lesser angulation and torqued to find the optimal “angle of attack,” enabling the microcatheter to be pushed more distally into the desired branch. This feature allows the wire to bend most 0.017 microcatheter tips, and even some 0.021 and 0.027 microcatheters, eliminating the need for a dedicated articulated microcatheter. This feature can be useful to avoid getting stuck in the ophthalmic artery or PCOM origin when passing a large aneurysm neck or when distal support or aspiration catheters are being navigated across the siphon of the ICA. A technical video on the use of Artiria SmartGUIDE microwire showing the wire's dynamic features in vivo has been recently published.^8^

### Recommendations

SmartGUIDE enhances navigation capabilities, allowing access to challenging distal, angulated target vessels. The handle acts as an extended torque device, enabling full 360° tip deflection while simultaneously pushing and pulling the wire single-handedly. Care should be taken when clamping the handle on the proximal 40 cm of the wire to avoid overtightening, which can cause kinking and damage the wire. In very tortuous anatomy, tip deflection may experience delays, and tension can build up within the system, which can be relieved by gently pushing the wire back and forth to release excessive built-up stress. A key feature to get used to is the area between the handle and the Y-connector of the microcatheter, which is typically less rigid than most traditional 0.014-inch wires but still provides support for stiffer materials such as balloons and stents. This portion should be handled carefully to prevent kinking or damage. First-time users are encouraged to thoroughly practice with 3D-printed silicone models to familiarize themselves with the wire's features before using it in patients.

### Study limitations

This early feasibility study is mainly limited by its retrospective, single arm design and relatively small cohort. Case diversity seems to demonstrate versatility and safety, but there was no control group or head-to-head comparison with competitors. Therefore, this study is not aimed to demonstrate superiority against conventional microwires. Metrics such as time to target access and the fluoroscopy time used per case were not systematically recorded, as these were beyond the scope of this initial evaluation. Future prospective studies may be required to thoroughly evaluate procedural time, radiation dose, complications, costs and other secondary outcomes against the established wires.

## Conclusion

SmartGUIDE's 0.014-inch deflectable tip allows real-time wire and microcatheter shaping, enabling safe and efficient access across a range of neurovascular procedures. It minimizes the need for adjunctive devices and manual wire reshaping, potentially lowering procedural risk and cost. Compatibility with standard microcatheters and its ability to direct microcatheter tips without leading with the wire represent significant innovations. Further studies are warranted to confirm these preliminary findings.

## Supplemental Material

sj-docx-1-ine-10.1177_15910199251399461 - Supplemental material for Early experience with the SmartGUIDE: A new generation of in-situ deflectable 0.014-inch guidewire Supplemental material, sj-docx-1-ine-10.1177_15910199251399461 for Early experience with the SmartGUIDE: A new generation of in-situ deflectable 0.014-inch guidewire by Mohammad Al-Tibi, James Lord, Shah Islam, Federico Carpani, Eef J Hendriks, Emily Chung, Alexandre Boutet, Ronit Agid, Zeev Itsekzon Hayosh and Pascal J Mosimann in Interventional Neuroradiology

## Abbreviations

Abbreviation: Definition
ACOM: Anterior communicating artery
AVM: Arteriovenous malformation
BTO: Balloon test occlusion
CCF: Carotid-cavernous fistula
CT: Computed Tomography
CTA: CT Intracranial Angiography
DL: Double lumen
dAVF: Dural arteriovenous fistula
eTICI: Expanded thrombolysis in cerebral infarction
EVT: Endovascular thrombectomy
ICA: Internal carotid artery
IPS: Inferior Petrosal Sinus
MCA: Middle cerebral artery
MMA: Middle meningeal artery
mRR: Modified Raymond-Roy classification
mRS: Modified Rankin Scale
n-BCA: n-Butyl cyanoacrylate
Onyx: Ethylene-vinyl alcohol copolymer
PCA: Posterior cerebral artery
PCOM: Posterior communicating artery
PICA: Posterior inferior cerebellar artery
PTA: Percutaneous transluminal angioplasty
SCA: Superior cerebellar artery
SCC: Squamous cell carcinoma
SG: SmartGUIDE
SOV: Superior ophthalmic vein
P2Y12: Platelet aggregation inhibitor
MRI: Magnetic Resonance Imaging
MRA: Magnetic Resonance Angiography
TOF: Time of Flight
