# Supplementary material for: Early experience with the SmartGUIDE: A new generation of in-situ deflectable 0.014-inch guidewire
Source: Interv Neuroradiol. 2025 Nov 27:15910199251399461. Online ahead of print. doi: 10.1177/15910199251399461 (PMC12660130; doi:10.1177/15910199251399461)
Supplement: sj-docx-1-ine-10.1177_15910199251399461 - Supplemental material for Early experience with the SmartGUIDE: A new generation of in-situ deflectable 0.014-inch guidewire [file sj-docx-1-ine-10.1177_15910199251399461.docx]

**Supplemental material**

**Early experience with the SmartGUIDE: A New Generation of In-Situ Deflectable 0.014-Inch Guidewire.**

**Figure 1: Stent assisted coiling of bilobed ACOM aneurysm (Patient 5)**


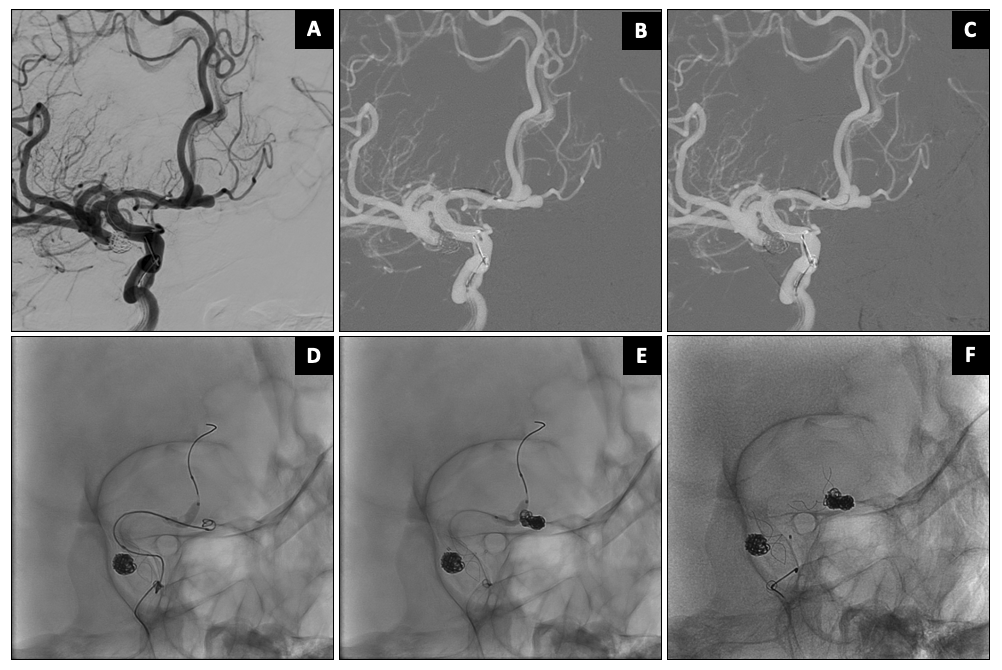


Caption: Stent assisted coiling of bilobed ACOM aneurysm (A). Functionality of SmartGUIDE wire was used to safely cannulate each lobe of the aneurysm using different wire configuration settings (B-C). With balloon protection through the ipsilateral A2, progressive aneurysm coiling was achieved (D-E). Braided stent was deployed through the balloon catheter for increased durability (F).
